# Bone Marrow-Derived NCS-01 Cells Advance a Novel Cell-Based Therapy for Stroke

**DOI:** 10.3390/ijms21082845

**Published:** 2020-04-19

**Authors:** John Brown, You Jeong Park, Jea-Young Lee, Thomas N. Chase, Minako Koga, Cesar V. Borlongan

**Affiliations:** 1 Center of Excellence for Aging and Brain Repair, Department of Neurosurgery and Brain Repair, University of South Florida College of Medicine, Tampa, FL 33612, USA; john182@mail.usf.edu (J.B.); youjeongpark@usf.edu (Y.J.P.); jeayoung@usf.edu (J.-Y.L.); 2 KM Pharmaceutical Consulting LLC, Washington, DC 20006, USA; tchase@chasetherapeutics.com (T.N.C.); mkoga@kmphc.com (M.K.)

**Keywords:** mesenchymal stem cells, cerebral ischemia, middle cerebral artery occlusion, regenerative medicine, interleukin-6, basic fibroblast growth factor, filopodia

## Abstract

Human mesenchymal stem cells have been explored for their application in cell-based therapies targeting stroke. Identifying cell lines that stand as safe, accessible, and effective for transplantation, while optimizing dosage, timing, and method of delivery remain critical translational steps towards clinical trials. Preclinical studies using bone marrow-derived NCS-01 cells show the cells’ ability to confer functional recovery in ischemic stroke. Coculturing primary rat cortical cells or human neural progenitor cells with NCS-01 cells protects against oxygen-glucose deprivation. In the rodent middle cerebral artery occlusion model, intracarotid artery administration of NCS-01 cells demonstrate greater efficacy than other mesenchymal stem cells (MSCs) at improving motor and neurological function, as well as reducing infarct volume and peri-infarct cell loss. NCS-01 cells secrete therapeutic factors, including basic fibroblast growth factor and interleukin-6, while also demonstrating a potentially novel mechanism of extending filopodia towards the site of injury. In this review, we discuss recent preclinical advancements using in vitro and in vivo ischemia models that support the transplantation of NCS-01 in human stroke trials. These results, coupled with the recommendations put forth by the consortium of Stem cell Therapeutics as an Emerging Paradigm for Stroke (STEPS), highlight a framework for conducting preclinical research with the ultimate goal of initiating clinical trials.

## 1. Introduction

Ischemic stroke poses as one of the leading causes of death and disability in the modern world [1]. The current treatment for stroke involves reperfusion therapy such as tissue plasminogen activator (tPA) or mechanical thrombectomy (MT). Tissue plasminogen activator (tPA) represents the sole FDA-approved drug for treating stroke but must be intravenously administered within 4.5 h to be effective [2,3]. This narrow time window disqualifies most patients and leads to only 3% of ischemic stroke patients benefiting from tPA treatment [4]. Therefore, limited treatment options and the short therapeutic window warrant investigating novel modalities for treating stroke outside this window [5,6]. 

The neuroinflammatory response that arises from an ischemic event plays a significant role in stroke pathology [7,8,9]. The blood–brain barrier (BBB) manifests as a dynamic, rigorously regulated border that modulates the exchange of ions, molecules, and cells between the central nervous system and surrounding blood [10]. A cascade of mechanisms involving the immune-inflammatory, thrombotic, and fibrinolytic pathways following ischemic stroke contributes largely to the damage of the BBB, which leads to the loss of tight junction integrity, increased permeability, edema, brain damage, and ultimately neurological dysfunction [11,12,13]. Outside of ischemic stroke, targeting these inflammatory pathways renders therapeutic benefits to the injured brain [14,15]. One approach that has emerged as an effective experimental treatment for stroke involves cell-based regenerative medicine. Mesenchymal stem cells (MSCs), which are nontumorigenic and easily accessible from donor tissue sources, stand as a promising candidate for poststroke cell therapy [16,17,18,19,20]. The functional recovery produced by MSC transplantation may be due to the cells’ release of trophic or anti-inflammatory factors instead of the initial concept of cell replacement mechanism [21,22,23]. This updated perspective better aligns with MSC’s in vivo role in secreting immunomodulatory and trophic mediators in response to injury or inflammation in the ischemic tissues [24,25]. When exogenous MSCs are transplanted in ex vivo and in vivo models of stroke, they secrete these immunomodulatory mediators, which have been found to attenuate the damage caused by neuroinflammation [8,17,26,27].

Although preclinical studies provide ample support for the use of MSCs in human clinical trials, two clinical trials using MSCs have failed to translate these findings in human stroke [28,29]. Intravenous administration of autologous bone marrow MSCs 4 weeks after stroke showed functional improvements at 3 and 6 months post treatment, but these effects diminished by 12 months [28]. Aside from showing that MSCs remain safe for transplantation, the outcome of these clinical trials highlights the importance of (1) recognizing and addressing translational gaps and (2) taking rigorous measures in the preclinical stage to optimize treatment dosage, target patient population, delivery method, and timing [30]. These concerns are also raised in the most recent preclinical research guidelines put forth by the Stem cell Therapeutics as an Emerging Paradigm for Stroke (STEPS) consortium [31].

**Figure 1:**
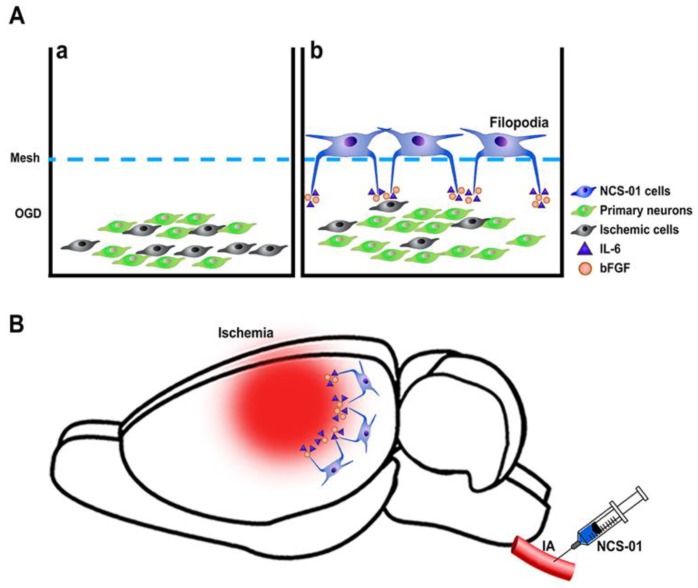
NCS-01 cells rescue neurons (**A**) in vitro study, NCS-01 cells used filopodia to modulate a long-distance mechanism of rescuing primary rat cortical neurons exposed to oxygen glucose deprivation (OGD). (**a**) Primary rat neurons subjected to OGD alone had more ischemic cells. (**b**) Primary rat neurons subjected to OGD and cocultured with NCS-01 cells demonstrated a significant increase in survival rate. NCS-01 cells grew filopodia toward the primary neurons. This implicates a novel rescue mechanism in which NCS-01 cells use cytokines interleukin-6 (IL-6), basic fibroblast growth factor (bFGF), and filopodial extensions to mediate the rescue of neurons from ischemic environments. (**B**) In vivo study, NCS-01 cells were injected via intracarotid artery (ICA) resulting in reduced infarct area, less peri-infarct cell loss, and improved motor and neurological behaviors.

Transplantation of NCS-01 cells in stroke models may help ameliorate some of these gaps in translation. In July 2019, NSC-01 cells received FDA approval for clinical application of intracarotid (ICA) transplantation in ischemic stroke patients [32]. Here, we review the latest findings of NCS-01 transplantation in in vitro and in vivo models of ischemic stroke that elucidate the effect of dosage, timing, delivery method, and the potential mechanism on its therapeutic effects (Figure 1).

## 2. NCS-01 Cells In Vitro

In vitro studies demonstrate that NCS-01 cells dose-dependently protect cocultured primary rat cortical neurons and astrocytes subjected to oxygen-glucose deprivation (OGD), although an increase of NCS-01 cells over a threshold ratio of 1:1 did not significantly increase host cell survival [32]. This type of exogenous benefit concurs with a growing body of evidence that cell-based therapy provides a type of therapeutic trophic chaperone support to injured ischemic tissue [23,26]. Exogenous therapy presents a more integrative approach toward a broader number of cells affected by cerebral ischemia [33,34]. Bone marrow-derived stem cells such as NCS-01 contain endogenous anti-inflammatory cytokines including interleukin-6 (IL-6), and more proactively secrete them when encountering other cells subjected to ischemia [35]. It is important to note that discrepancies exist throughout literature regarding the role of IL-6 expression in inflammation. While IL-6 typically confers proinflammatory effects [36,37,38], its role in the brain is more diverse and dependent on the region and timing of expression [39]. For example, IL-6 has been implicated in poststroke social isolation in mice, and IL-6 levels drop in the brain and increase in the plasma after isolation [40]. Blocking the isolation-induced loss of brain IL-6 leads to improved outcomes after stroke, suggesting that IL-6 signaling differs more in the brain than in the peripheral tissues [40]. IL-6 also affects the balance between M1 and M2 types phenotype of macrophages—important determinants of alternative microglial activation and polarization [40]. Although the role of IL-6 in inflammation has not yet been fully elucidated, its effects are diverse in the brain and may be therapeutic in the setting of neuro-inflammation following ischemic stroke [39,40,41,42].

Another consequence of OGD that contributes to neuronal death and inflammation involves the dysfunction of the mitochondria [43,44]. Mitochondrial perturbation impairs oxidative metabolism and reduces the production of total adenosine triphosphate (ATP) while increasing the production of reactive oxygen species (ROS) [45]. Interestingly, stem cells convey healthy mitochondria to compromised ischemic cells [44]. Stem cell therapy-mediated repair of mitochondria may also help reduce inflammation endemic to stroke [46]. NCS-01 cells not only increase cell viability but also double the mitochondrial activity when cocultured with neurons and astrocytes subjected to OGD in a 4:1 ratio of NCS-01 cells to host cells [32].

In vitro studies also suggest that NCS-01 cells demonstrate a potentially novel mechanism through which their filopodia may exert therapeutic effects under stroke conditions. To investigate the mechanism of action behind NCS-01 filopodia, primary rat cortical cells exposed to OGD were cocultured with NCS-01 cells at various distances ranging from 0 mm to 2.04 mm [32]. Imaging studies revealed the presence of cadherin-positive filopodia extending from NCS-01 cells to the injured cells. Maximal rescue of cell viability and mitochondrial activity in cells entails a direct contact with NCS-01 cells when compared to cells treated with non-stem cells [32]. The degree of rescue negatively correlates with the distance between cells exposed to OGD and the NCS-01 cells. However, the two farther distances (1.92 and 2.04 mm) still display significantly improved cell viability and mitochondrial activity when compared to the non-stem cell treatment [32]. These results suggest that direct contact stands as optimal, but indirect long-distance rescue via filopodia may be possible.

To tease apart the effects of IL-6, bFGF, and NCS-01-derived filopodia in cell rescue, other studies were performed on various host cells, including primary cortical neurons, primary rat astrocytes, and primary rat endothelial progenitor cells (EPCs). Each cell type was subjected to OGD and treated with either (1) cell media only (control), (2) IL-6 + bFGF only, (3) NCS-01 cells only, or (4) a combination of IL-6 + bFGF + NCS-01 cells. All treatment groups exhibit improved mitochondrial activity compared to the control group, with the greatest activity seen in groups treated with NCS-01 cells only and IL-6 + bGFG only. Among the different host cell types, neurons display the greatest recovery, in that NCS-01 cell treatment renders significantly better therapeutic outcomes than IL-6 + bGFG. However, IL-6 + bFGF treatment and NCS-01 treatment afford comparable rescue for astrocytes and EPCs. Interestingly, the combination treatment groups (IL-6 + bFGF + NCS-01 cells) perform significantly worse than when each treatment was given alone for neurons and EPCs but not for astrocytes. Furthermore, in all three neuronal host cell lines, filopodia formation accompanies both the NCS-01 cell only treatment and the combination of IL-6 + bFGF + NCS-01 cells treatment, indicating that filopodia formation correlates with improved cell viability and mitochondrial activity. These results suggest that NCS-01 cell’s therapeutic effects stem from the release of IL-6 and bFGF, and filopodia formation. Further manipulation of cytokine release, and facilitating or reducing filopodia formation, may elucidate more mechanisms of brain repair and its application to cellular therapy [47].

Filopodia formation participates in neuroprotection by Rho-GTPase kinase inhibition on organotypic hippocampal slices subjected to ischemia [48]. Similarly, overexpression of CD44, a signaling molecule involved in the transendothelial migration of lymphocytes by neural precursor cells, promotes the elongation and spread of filopodia in vitro when seeded on laminin and may facilitate the invasion of certain perivascular sites [49]. Additionally, high-mobility group box 1 (HMGB1), a nonhistone nuclear DNA-binding protein, induces NSC filopodia formation and upregulates expression of one of its receptors called RAGE (receptor for advanced glycation end products) [50]. Cell motility and filopodia formation may be aided by adhesion molecules and transcription factors, and understanding their roles may improve the therapeutic outcome of NCS-01 cells in stroke.

## 3. NCS-01 Cells In Vivo

Optimizing NCS-01 cell dosage, delivery method, target population, and timing of transplantation in rat ischemic stroke models remains critical to advancing translation. Despite solid preclinical evidence, clinical trials using other MSCs result in modest functional improvements in patients, highlighting the importance of optimization studies [7]. To investigate NCS-01 cell dosage on treatment efficacy in vivo, rats subjected to 1 h transient middle cerebral arterial occlusion (MCAO) received either saline or quantities of 7.5 × 10^5^, 7.5 × 10^6^, or 3.75 × 10^7^ NCS-01 cells in a set concentration of 7.5 × 10^6^ NCS-01 cells/mL via the ICA method of delivery [32]. Consistent with therapeutic outcomes in other animals transplanted with MSCs [51], all three treatment dosages reduce infarct volume and neurological deficit scores by 25% and 50% of saline controls [32]. The lack of significant difference between the three treatment groups using NCS-01 cells suggests that the concentration of 7.5 × 10^6^ NCS-01 cells per mL at a dose as low as 7.5 × 10^5^ cells per 0.1 mL corresponds to the minimum effective in vitro dose, while the highest dose of 3.75 × 10^7^ NCS-01 cells per 5 mL dose still remains well tolerated in animals for up to 28 days post MCAO [32]. These observed efficacy readouts with NCS-01 cells corroborate therapeutic effects of MSC transplantation following an ischemic event [52,53]. 

To find the minimum effective in vivo dose, MCAO rats received either 7.5 × 10^5^ NCS-01 cells in 0.1 mL, 2.5 × 10^5^ NCS-01 cells in 0.03 mL, 7.5 × 10^4^ NCS-01 cells in 0.1 mL, or 7.5 × 10^6^ rat fibroblasts in 1 mL. All three treatment groups displayed significantly smaller infarct volumes than the fibroblast group. Moreover, animals transplanted with 2.5 × 10^5^ and 7.5 × 10^5^ NCS-01 cells exhibit significantly lower neurological deficit scores than those that received fibroblasts. Overall, the dose of 7.5 × 10^5^ NCS-01 per 0.1 mL robustly reduces infarct volume and improves neurological function [32]. 

Determining optimal delivery method of NCS-01 cells in rat MCAO models represents another critical factor for successful lab-to-clinic translation of NCS-01 cells. IV or ICA dose of 7.5 × 10^6^ NCS-01 cells in 1 mL promotes significant pathological and neurological recovery compared to saline administration [32]. However, ICA delivery of NCS-01 cells reduces infarct volume almost twice as much as IV-delivered cells, suggesting that administration through ICA rescues the ischemic brain more effectively than IV [32]. This is consistent with previous findings that MSCs injected intra-arterially coincide with functional recovery in cerebral stroke animals [54,55]. By taking the arterial route, donor cells can bypass filtering organs and directly migrate toward the middle cerebral artery and ischemic hemisphere. Although both delivery routes for NCS-01 cells decrease neurological deficit scores compared to saline administration, these results suggest ICA delivery of NCS-01 may produce better stroke outcomes than IV delivery in at least one aspect of brain damage.

To establish a target patient population, ICA delivery of NCS-01 cells in transient MCAO was compared to permanent MCAO. Consistent with other MSCs in MCAO rats [56], ICA delivery of NCS-01 cells in transient MCAO elicits better anti-inflammatory responses, tissue repair, and functional benefits, which predicts better results for stroke patients successfully revascularized by either thrombectomy or tPA. Furthermore, investigation of the therapeutic window shows that NCS-01 cell treatment remains effective when initiated 1 week after MCAO, but most effective when started 3 days or earlier [32]. These parameters are important to consider when establishing inclusion and exclusion criteria for future clinical trials. 

Based on the recommendations put forth by STEPS, safety assessment remains a critical focus in preclinical studies [31]. Bone marrow-derived stem cells, particularly MSCs, have been extensively evaluated for safety in both humans and animals [57,58,59,60,61]. NCS-01 cells, while exhibiting a robust response and broad therapeutic window, demonstrate no histological or behavioral side effects and have obtained FDA approval for stroke clinical trials. Furthermore, dose escalation studies using 5 × 10^7^ cells in 5 mL, 1 × 10^7^ cells in 5 mL, 5 mL of saline, or no infusion in rat MCAO suggest that ICA administration of NCS-01 cells may be just as safe as no infusion, when assessed by cerebral brain flow [32]. 

## 4. Comparing NCS-01 Cells with Other Cell Lines

In vitro studies elucidate differences in treatment efficacy and rescue mechanisms between NCS-01 cells and other MSCs, such as the Li cell line [32,62]. MSCs’ secretion of trophic or anti-inflammatory factors, such as β-NGF, BDNF, VEGF, IGF-1, bFGF, and IL-6, confers their neuroprotective effects [32,63]. Comparing human neural progenitor cells, primary rat cortical neurons, and astrocytes subjected to OGD and cocultured with NCS-01 or Li cells shows that both NCS-01 and Li cells rescue against cell death and increase bFBF and IL-6 production, suggesting these two molecules stand as the main drivers behind the protective effects seen after treatment [32,62]. However, NCS-01 cells produce almost four times as much bFGF and IL-6 as Li cells, suggesting a significant phenotypic difference despite both cell lines being characterized as MSCs [32]. 

In vivo studies also reveal that NCS-01 cells provide significantly better treatment efficacy than Li cells. MCAO rats treated with NCS-01 cells see a 60% reduction in their infarct volume and 50% reduction of neurological deficits within 7 days [32]. With therapeutic effects unmatched by Li cells administered at the same dose, NCS-01 proves to be a unique donor cell population. 

## 5. Conclusions

Ischemic stroke persists as one of the top contributors of death and disability worldwide, which emphasizes an increasingly urgent need for effective treatments in today’s aging population. The transplantation of MSCs shows tremendous promise as an experimental treatment option for stroke [28,29,64,65]. Preclinical findings with NCS-01 cells demonstrate safety, treatment efficacy, and a potentially novel filopodia mechanism for regenerative medicine in stroke. Administration of NCS-01 cells improves neurologic function by reducing infarct volume and cell death in the peri-infarct region. In vitro studies suggest these beneficial effects may stem from the secretion of therapeutic molecules such as bFGF and IL-6, as well as the formation of filopodia. Moreover, additional trophic and anti-inflammatory factors may be secreted by NCS-01 cells in vivo since host environment influences secretion [27,32]. In vivo studies indicate the optimal dose is 7.5 × 10^6^ cells/mL delivered via ICA within 3 days post stroke, although administration 7 days after stroke still yields significant therapeutic results.

Collectively, these studies support NCS-01 transplantation as a potential clinical approach in treatment of stroke. Before these findings can be translated to clinical trials, further investigation on the differences in anatomical, circulatory, and physiological systems between animals and humans may aid in translating on the optimal transplant regimen to clinical applications [31]. In particular, future studies may consider using larger animal models of the central nervous system such as nonhuman primates to better mimic human clinical pathologies and further optimize treatment parameters, including dosage, treatment timing, and the delivery route of NCS-01 cells. In parallel, incorporation of comorbidities commonly associated with human stroke, such as aging and hypertension, in animal models will closely approximate the clinical scenario of stroke, thereby representing a more stringent platform for testing the safety and efficacy profile of stem cells. 

In July 2019, FDA approved NCS-01 cell transplantation in ischemic stroke patients and a multicenter clinical trial study evaluating its safety started in February 2020 (ClinicalTrials.gov [https://clinicaltrials.gov/ct2/show/NCT03915431]). As stated in the recommendations put forth by STEPS, preclinical efficacy studies specifying target patient profiles should be conducted parallel to phase I/IIa clinical trials in order to identify populations to be considered in subsequent phase IIb/III clinical trials [31]. The preclinical in vitro and in vivo data on optimal NCS-01 cell dosage, delivery method, target population, timing, and safety presented in this review are critical parameters to consider when designing efficacy-driven clinical trials in the future, and represent a robust framework for conducting preclinical research with the ultimate goal of translation to human clinical trials. 

## Abbreviations

The following abbreviations are used in this manuscript:

tPA	tissue plasminogen activator
MT	mechanical thrombectomy
BBB	blood–brain barrier
MSCs	mesenchymal stem cells (MSCs)
STEPS	Stem cell Therapeutics as an Emerging Paradigm for Stroke
OGD	oxygen glucose deprivation
IL-6	interleukin-6
bFGF	basic fibroblast growth factor
IV	intravenous
ICA	intracarotid artery
ATP	adenosine triphosphate
ROS	reactive oxygen species
EPCs	endothelial progenitor cells
HMGB1	high-mobility group box 1
RAGE	receptor for advanced glycation end products
MCAO	middle cerebral arterial occlusion
